# Small molecule inhibitor of TGF-β signaling enables robust osteogenesis of autologous GMSCs to successfully repair minipig severe maxillofacial bone defects

**DOI:** 10.1186/s13287-019-1281-2

**Published:** 2019-06-13

**Authors:** Anyuan Shi, Aerali Heinayati, Dongyu Bao, Huifen Liu, Xiaochen Ding, Xin Tong, Liudi Wang, Bin Wang, Haiyan Qin

**Affiliations:** 1 Department of Dental Implantology, Nanjing Stomatological Hospital, Medical School of Nanjing University, 30 Zhongyang Road, Nanjing, 210008 China; 2 Nanjing Key Laboratory, Nanjing Stomatological Hospital, Medical School of Nanjing University, Nanjing, 210093 China; 3 Clinical Stem Cell Center, the Affiliated Drum Tower Hospital of Nanjing University Medical School, 321 Zhongshan Road, Nanjing, 210008 China

**Keywords:** GMSCs, SB431542, TGF-β signaling, Osteogenic differentiation, BMP, Bone defect

## Abstract

**Background:**

Clinically, for stem cell-based therapy (SCBT), autologous stem cells are considered better than allogenic stem cells because of little immune rejection and no risk of communicable disease infection. However, severe maxillofacial bone defects restoration needs sufficient autologous stem cells, and this remains a challenge worldwide. Human gingival mesenchymal stem cells (hGMSCs) derived from clinically discarded, easily obtainable, and self-healing autologous gingival tissues, have higher proliferation rate compared with autologous bone marrow mesenchymal stem cells (hBMSCs). But for clinical bone regeneration purpose, GMSCs have inferior osteogenic differentiation capability. In this study, a TGF-β signaling inhibitor SB431542 was used to enhance GMSCs osteogenesis in vitro and to repair minipig severe maxillofacial bone defects.

**Methods:**

hGMSCs were isolated and cultured from clinically discarded gingival tissues. The effects of SB431542 on proliferation, apoptosis, and osteogenic differentiation of hGMSCs were analyzed in vitro, and then, SB431542-treated hGMSCs composited with Bio-Oss® were transplanted into immunocompromised mice subcutaneously to explore osteogenic differentiation in vivo. After that, SB431542-treated autologous pig GMSCs (pGMSCs) composited with Bio-Oss® were transplanted into circular confined defects (5 mm × 12 mm) in minipigs maxillary to investigate severe bone defect regeneration. Minipigs were sacrificed at 2 months and nude mice at 8 weeks to retrieve specimens for histological or micro-CT or CBCT analysis. Effects of SB431542 on TGF-β and BMP signaling in hGMSCs were investigated by Western Blot or qRT-PCR.

**Results:**

One micromolar of SB431542 treatment induced a robust osteogenesis of hGMSCs in vitro, without adverse effect on apoptosis and growth. In vivo, 1 μM SB431542 treatment also enabled striking osteogenesis of hGMSCs subcutaneously in nude mice and advanced new bone formation of pGMSCs in minipig maxillary bone defect model. In addition, SB431542-treated hGMSCs markedly increased bone-related proteins expression, and *BMP2* and *BMP4* gene expression. Conversely, SMAD3 protein-dependent TGF-β signal pathway phosphorylation was decreased.

**Conclusions:**

Our study show that osteogenic differentiation of GMSCs treated with TGF-β signaling inhibitor SB431542 was increased, and SB431542-treated autologous pig GMSCs could successfully repair minipig severe maxillofacial bone defects. This preclinical study brings about a promising large bone regeneration therapeutic potential of autologous GMSCs induced by SB431542 in clinic settings.

**Electronic supplementary material:**

The online version of this article (10.1186/s13287-019-1281-2) contains supplementary material, which is available to authorized users.

## Background

The repair of large-volume skeletal defect remains one of most difficult medical challenges worldwide [1–3]. In contrast to other tissues, a large-scale bone defect has almost lost its self-healing ability, and optimal solutions are relative poor. The current therapeutic strategies include the application of allogeneic/autologous bone and prosthetic substitute, to achieve the goal of reconstructing the lost bone tissue [4, 5]. However, the clinic outcomes of these approaches in complex maxillofacial bone defects are limited and highly associated with a series of complications including immunologic rejection and infection. Even though the transplantation of autologous bone tissue is considered the gold standard for treating severe bone defects it has some downsides such as chronic pain, bleeding inflammation, donor site morbidity, scaring, and insufficient bone tissue [6]. Thus, developing alternative strategies of repairing severe maxillofacial bone defects is a pressing task to meet the increasing clinical need.

Stem cell-based therapy (SCBT) brings about great potential for therapeutic application in severe maxillofacial bone defects [7, 8]. Mesenchymal stem cells (MSCs) which have osteogenic differentiation capability and immunoregulatory function are considered ideal stem cells for bone regeneration. [9–11]. Autologous cells are considered better than allogenic cells because of little immune rejection and no risk of communicable disease infection [12, 13]. For clinically bone reconstruction and regeneration purpose, osteogenic differentiation potential of human bone marrow mesenchymal stem cells (hBMSCs) is more competitive compared to other types of autologous MSCs [14–17]. But bone marrow aspirate is an invasive method, with a limited amount of BMSCs, and is not easily accepted for many people. Human gingival tissue-derived MSCs (hGMSCs) are among the best autologous cell source for bone defect repairing because they are derived from clinically discarded, easily obtainable gingival tissue by extraction of teeth, surgical crown lengthening, and periodontal surgeries etc., without additional damage to patients. Besides, hGMSCs also could be obtained from healthy gingival tissue which has remarkable self-healing and regenerative potential after injury or resection, without any evidence of scar formation. In addition, the easily cultured hGMSCs exhibit homogeneity in primary and maintain high proliferation rate, differentiation capability, stable phenotype, karyotype, and telomerase activity in long-term cultures [18–20]. Therefore, a large number of autogenous needed MSCs can be obtained from human gingival tissues in a short of time for cell therapy in clinic. But osteogenic differentiation potential of hGMSCs is still not enough compared to hBMSCs [14]. Thus, there is a high impetus for developing an effective approach to enhance osteogenic differentiation if GMSCs are subjected to restore severe maxillary bone defect.

A variety of key signaling pathways orchestrated to regulate skeletal development, homeostasis, turnover, and regeneration [21, 22]. The transforming growth factor beta (TGF-β) plays a crucial role in bone physiology such as osteogenic differentiation and bone regeneration [2, 23]. Small molecule SB431542, as a potent and specific TGF-β type I receptor inhibitor, had been reported to potentiate endogenous bone regeneration [2]. In addition, SB431542 treatment shows a potential to enhance osteogenic differentiation of cord blood-derived unrestricted somatic stem cells and periodontal ligament cells depending on different context [24, 25]. However, whether SB431542 could be used to promote GMSCs osteogenesis is unknown. In this study, we used SB431542 treatment to promote robust osteogenic differentiation of human and pig GMSCs in vitro and further proved that SB431542 treatment also promoted new osteogenesis of hGMSCs after subcutaneous implantation in nude mice and bone regeneration after transplanting pig autologous GMSCs in minipig maxillary bone defects model, indicating this new strategy is promising in repairing severe maxillofacial bone defects in clinic settings.

## Methods

### Isolation and culture of hGMSCs

Primary hGMSCs were isolated from clinically discarded gingival tissues of informed donors approved by the Ethics Committee of Nanjing Stomatological Hospital (permit number: 2015NL-002) (5 donors: 2 women, aged 18 and 20 years, and 3 men, aged 16 years,18 years, and 21 years). In brief, the gingival tissues were mechanically minced into small pieces (< 1 mm^3^) and seeded in a 10-cm culture plate (Corning, NY, USA) with culture media containing α-modified minimum essential medium (α-MEM; Gibco, Grand Island, NY, USA) supplemented with 15% fetal bovine serum (FBS; Gibco), 100 mM l-ascorbic acid 2-phosphate (Sigma-Aldrich, St. Louis, MO), 100 U/ml penicillin, and 100 mg/ml streptomycin (Sigma-Aldrich) in a humidified incubator at 37 °C and 5% CO_2_, as previously described [26]. The culture medium was changed every 3 days. hGMSCs were detached with 0.25% trypsin/EDTA (Gibco) and passaged when they had grown to 80% confluence. After the first passage, the hGMSCs obtained from 5 donors were pooled and cultured together for avoiding individual heterogeneity. Then, the identified cells at passage 3 were used for subsequent experiments in vitro and in vivo.

### Colony-forming unit assay of hGMSCs

Single-cell suspensions of cells (1 × 10^3^ cells) within ɑ-MEM containing 15% FBS were seeded into 10-cm culture plates and then incubated at 37 °C in 5% CO_2_. After 14 days of cultivation, cultures were fixed with 4% formalin, and then stained with 1% toluidine blue (Sigma-Aldrich).

### Cell viability assay of hGMSCs

About 1 × 10^5^ cells were cultured in 35-mm culture plate for cell viability assay. At 70–80% confluency, the cells were treated with various concentrations of SB431542 (0, 0.1, 1, 10 μM, Selleck chemicals, USA) for 24 h, respectively. Then, detached single-cell suspension with PBS solution and the live cells were counted by cell counter (Nexcelom) stained with 0.4% trypan blue solution (Sigma-Aldrich). Experiments were repeated three times using the same source of cells.

### Flow cytometry analysis of hGMSCs

For identification of MSC phenotype, approximately 5 × 10^5^ hGMSCs were incubated with phycoerythrin (PE) or fluroisothiocyanate (FITC)-conjugated monoclonal antibodies for human CD44, CD73, CD90, CD105, CD11b, CD19, CD34, CD45, HLA-DR, and HLA-DQ (BD Biosciences, NJ, USA) for 30 min at 4 °C according to the manufacturer’s recommendation. Experiments were repeated three times using the same source of cells. Cells were analyzed using a FACScan system (BD FACSAria™; NJ, USA), and Flow cytometry data were analyzed with FACS software.

### Apoptosis assay of hGMSCs

About 1 × 10^5^ cells were cultured in 35-mm culture plate for apoptosis assay. At 70–80% confluency, the cells were treated with various concentrations of SB431542 (0, 0.1, 1, 10 μM, Selleck chemicals, USA) for 24 h or 48 h. Then, single-cell suspension with PBS solution was prepared following the manufacturer’s protocol of ANNEXIN V-FITC/PI Apoptosis Detection Kit (Solarbio, Beijing, China). Rates of apoptosis were analyzed with FACScan system, and the data were analyzed with FACS software. Experiments were repeated three times using the same source of cells.

### Cell growth assay of hGMSCs

The proliferation of cells was analyzed using a WST-8 Cell Counting Kit-8 (CCK-8; Beyotime, Shanghai, China). Cells (2 × 10^3^) suspended in α-MEM medium (200ul) containing 15% fetal bovine serum were seeded in 96-well plates with SB431542 (0, 0.1, 1, 10 μM) and incubated for 1, 2, 3, 4, 5, 6, and 7 day, respectively. CCK-8 solution (20ul) was added to each well, and the cultures were incubated at 37 °C for 3 h. Absorbance at 450 nm was measured using an immunoreader. Four parallel replicates of each sample at each time point were prepared during this cell growth assay. Experiments were repeated three times using the same source of cells.

### Comparison of osteogenic differentiation between hBMSCs and hGMSCs in vitro

hGMSCs and hBMSCs which were provided by Clinical Stem Cell Center, The Affiliated Drum Tower Hospital of Nanjing University Medical School, at passage 3, were cultured in osteogenic induction medium at 100% confluency in a six-well plate. The osteogenic induction medium was α-MEM containing 15% FBS, 0.1 mM l-ascorbic acid 2-phosphate (Sigma-Aldrich), 100 U/ml penicillin, and 100 mg/ml streptomycin (Gibco), 1.8 mM KH_2_PO_4_ (Sigma-Aldrich), and 10^−8^ M dexamethasone sodium phosphate (Sigma-Aldrich). The culture media was changed every 3 days, and MSCs with induction medium were maintained for 21 days. The results were analyzed using Alizarin Red staining (Sigma-Aldrich). For quantification of Alizarin Red staining, mineralized nodules stained were faded with 10% cetylpyridinium chloride (Sigma-Aldrich) for 20 min at the room temperature. Absorbance at 562 nm of the supernatant was measured using an immunoreader in triplicate. Experiments were repeated three times using the same source of cells.

### SB431542-treated GMSCs osteogenic differentiation in vitro

hGMSCs at 100% confluency in six-well plate were cultured in osteogenic induction medium which was α-MEM containing 15% FBS, 0.1 mM l-ascorbic acid 2-phosphate (Sigma-Aldrich), 100 U/ml penicillin and 100 mg/ml streptomycin (Gibco), 1.8 mM KH_2_PO_4_ (Sigma-Aldrich), and 10^−8^ M dexamethasone sodium phosphate (Sigma-Aldrich). The culture media were changed every 3 days. hGMSCs induction with osteogenic induction medium in the presence or absence of SB431542 (0.1 and 1 μM) were maintained for 21 days. Besides, Recombinant Human Noggin was also added to induce medium with SB431542 to explore the interactions between the bone morphogenetic protein (BMPs) and SB431542. Firstly, hGMSCs were induced by medium in the presence of Noggin (250 ng/mL, Beyotime) from day 0 to day 3. Then, SB431542 (1 μM) was added into the osteogenic induction medium with noggin from day 4 to day 21. In addition, osteogenic differentiation of pig gingival mesenchymal stem cells (pGMSCs) treated with SB431542 in vitro are the same as hGMSCs. The results were analyzed using Alizarin Red staining (Sigma-Aldrich). Experiments were repeated three times using the same source of cells.

### Western blot assay

Proteins from cells were lysed with a RIPA lysis buffer containing 1 mM PMSF (Beyotime) referring to the manufacturer’s instruction. Then, the concentration was measured by BCA Protein Assay Kit (Beyotime). The proteins were separated by 10% sodium dodecyl sulfate (SDS) polyacrylamide gels and transferred to polyvinylidene difluoride membranes (Sigma-Aldrich). The membranes were blocked in 6% skim milk for 2 h and then incubated with primary antibodies at 4 °C overnight and finally with the secondary antibody (1:6000; Bioworld, Nanjing, China) for 1 h. The following primary antibodies were used: anti-GAPDH (1:6000; Proteintech™, WuHan, China), anti-RUNX2 (1:1000; Cell Signaling Technology, MA, USA), anti-OPN (1:1000, Proteintech™), anti-ALP (1:2000; Proteintech™), anti-COLLAGEN type I (1:2000;Proteintech™), anti-SMAD2(1:1000; Cell Signaling Technology), anti-phosphorylation SMAD2 (1:1000; Beyotime), anti-SMAD3 (1:1000; Beyotime), anti-phosphorylation SMAD3(1:1000; Beyotime),anti-ERK1/2(1:2000; Proteintech™), anti-phosphorylation ERK1/2(1:1000; Beyotime), anti-JNK (1:3000; Proteintech™), anti-phosphorylation JNK (1:1000; Beyotime), anti-P38(1:500; Proteintech™), and anti-phosphorylation P38(1:1000; Beyotime). The visualization of the protein bands was used with enhanced chemiluminescence kit (Beyotime).

### Quantitative real-time polymerase chain reaction (qRT-PCR)

Total RNA from hGMSCs, which were treated with uninduced, osteogenic induced, or osteogenic induced with SB431542 medium, was extracted with the trizol reagent kit (Invitrogen, Carlsbad, CA, USA). And cDNA was synthesis with Hiscript RT supermix kit (Vazyme; Nanjing, China) according to the manufacturer’s instructions. cDNA amplification was used by SYBR qPCR master mix (Vazyme), and qRT-PCR was performed by ABI QuantStudio™ 6 Flex system (Thermo Fisher Scientific, MASS, USA). All qRT-PCR reactions were performed in triplicate in each experiment. The BMPs genes (*BMP2* and *BMP4*) forward and reverse primers are listed in Additional file 1: Table S1. The results were analyzed using the 2^−ΔΔ*ct*^ method and normalized by *β-ACTIN*.

### Animals used in transplantation in vivo

All protocols involving animals were approved by the Animal Care and Use committee of The Affiliated Drum Tower Hospital of Nanjing University Medical School (permit number: NJGLYY2018010001), which were proposed as a randomized, controlled experimental ones. Three male Guangxi Bama minipigs, 10 months old and weighing 45–55 kg, were obtained from Guangxi. The BALB/c immunocompromised mice (male, aged 4–5 weeks, *n* = 4) were purchased from Vital River Laboratories (Beijing).

### Ectopic osteogenesis of hGMSCs in nude mice

hGMSCs at passage 3 were cultured in complete medium with or without 1 μM SB431542 for 48 h, then digested and seeded on the scaffolds Bio-Oss®, anorganic bovine bone scaffolds (diameter, 1–2 mm, Geistlich) for 90 min in the incubator. After nude mouse was anesthetized with 10% chloral hydrate (0.04 ml/10 g), three incisions in the dorsum skin (1 cm length) were made, then dorsal subcutaneous pockets were formed with incisions separated deeper. Subcutaneous pockets were for insertion of the following three groups randomly (four pockets each group): (1) treatment with a wax spoon amount of Bio-Oss®(Bio-Oss®), (2) treatment with the same amount of Bio-Oss® seeded with 2 × 10^6^ hGMSCs (Bio-Oss®/hGMSCs), (3) treatment with the Bio-Oss® containing 2 × 10^6^ hGMSCs and 1 μM SB431542(Bio-Oss®/hGMSCs/SB431542). The mice were sacrificed to retrieve specimens at 12 weeks.

### Bone regeneration in minipigs maxillary bone defects

The study of minipigs maxillary bone defect model was divided into three phases. Firstly, pig gingival MSC (pGMSCs) were isolated and cultured and then induced with osteogenic medium in vitro. Secondly, grafts of osteogenically induced pGMSCs were implanted into the maxillary defects in vivo. Finally, animals were sacrificed to retrieve specimens 2 months later. In brief, after preoperative evaluation with no abnormality of pigs, then anesthesia was induced with midazolam (0.2 mg/kg) and maintenance of anesthesia was used by propofol (4 mg/kg/h). Gingival tissues were collected from the three pigs under general anesthesia. And the isolation and culture of pGMSCs are the same as hGMSCs. The cells at passage 3 were used for the following experiments. Two intraosseous circular confined defects were created on each side of the maxillary in the second stage. The defects, measuring 12 mm in diameter and 5 mm in depth with periodontal probe, were made using a trephine [27–30]. There were altogether 12 defects generated in 3 mini pigs. The prepared defects on each side were filled with one of four grafting materials randomly (three defects each group): (a) autogenous bone blocks, (b) Bio-Oss®, (c) Bio-Oss®/autogenous pGMSCs (2 × 10^6^), and (d) Bio-Oss®/ autogenous pGMSCs (2 × 10^6^)/SB431542 (1 μM). Following graft placement, the absorbable collagen barrier membrane (Bio-Gide, Geistlich AG) was used to cover the defects. Then, undermining of mucoperiosteal tissue was performed to cover the graft material completely without tension. The animals were sacrificed by induction of deep anesthesia to retrieve specimens 2 months later.

### CBCT imaging re-establishment

The cone beam computed tomography (CBCT) images of nude mice and maxillary bone of minipigs were taken using a NewTom VG scanner (QR srl) at 0.20 mm solution. In the visualization of the 3D images of the allogeneic grafts, a new bone was created using Software NNT viewer.

### Micro-CT analysis

The specimens retrieved from minipigs at 2 months postsurgery were fixed in 4% paraformaldehyde for 48 h, then scanned using micro-CT (Hiscan M1000, Suzhou, China) (50kv,0.8 mA) at 50-μm resolution. The images were reconstructed using the ImageJ, which can distinguish the new bone from the old bone in this study. And the average CT value and bone density (BD) of a new bone in the bone defects were both calculated. Thresholding value of micro-CT for BD quantification is from 6533 to 10,096. The sample for CT value and BD quantification was three for each group.

### Histological analysis

The specimens retrieved from dorsal skin of immunocompromised mice and bone defect in minipigs were fixed in 4% paraformaldehyde for 48 h, decalcified with 5% EDTA for 45 days, embedded in paraffin blocks, and sectioned 2 mm thick for H&E and Masson staining. For H&E staining, the sections were stained in hematoxylin for 5 min, followed in eosin for 1 min. For Masson staining, the sections were conducted according to the Masson trichrome stain Kit manufacturer’s protocol (Sbjbio). Briefly, the sections were immersed in Weigert’s iron hematoxylin for 5 min, then plasma stain for 10 min, phosphomolybdic acid in distilled water for 1 min, and fiber stain for 1 min.

### Statistical analysis

All measurements were collected and expressed as mean ± standard deviation (SD) from at least three independent experiments. Data for these measurements were analyzed using Student’s *t* tests for paired comparisons or one-way analysis of variance (ANOVA) for multiple comparisons with Tukey’s multiple comparison test. GraphPad prism 6.0 (GraphPad Software, San Diego, CA, USA) software were utilized to analyze and demonstrate the statistical significance of the assays, and differences at *p* < 0.05 were considered statistically significant. The significance between groups was marked on the graphs.

## Results

### The phenotypic characteristics of hGMSCs

**Fig. 1:**
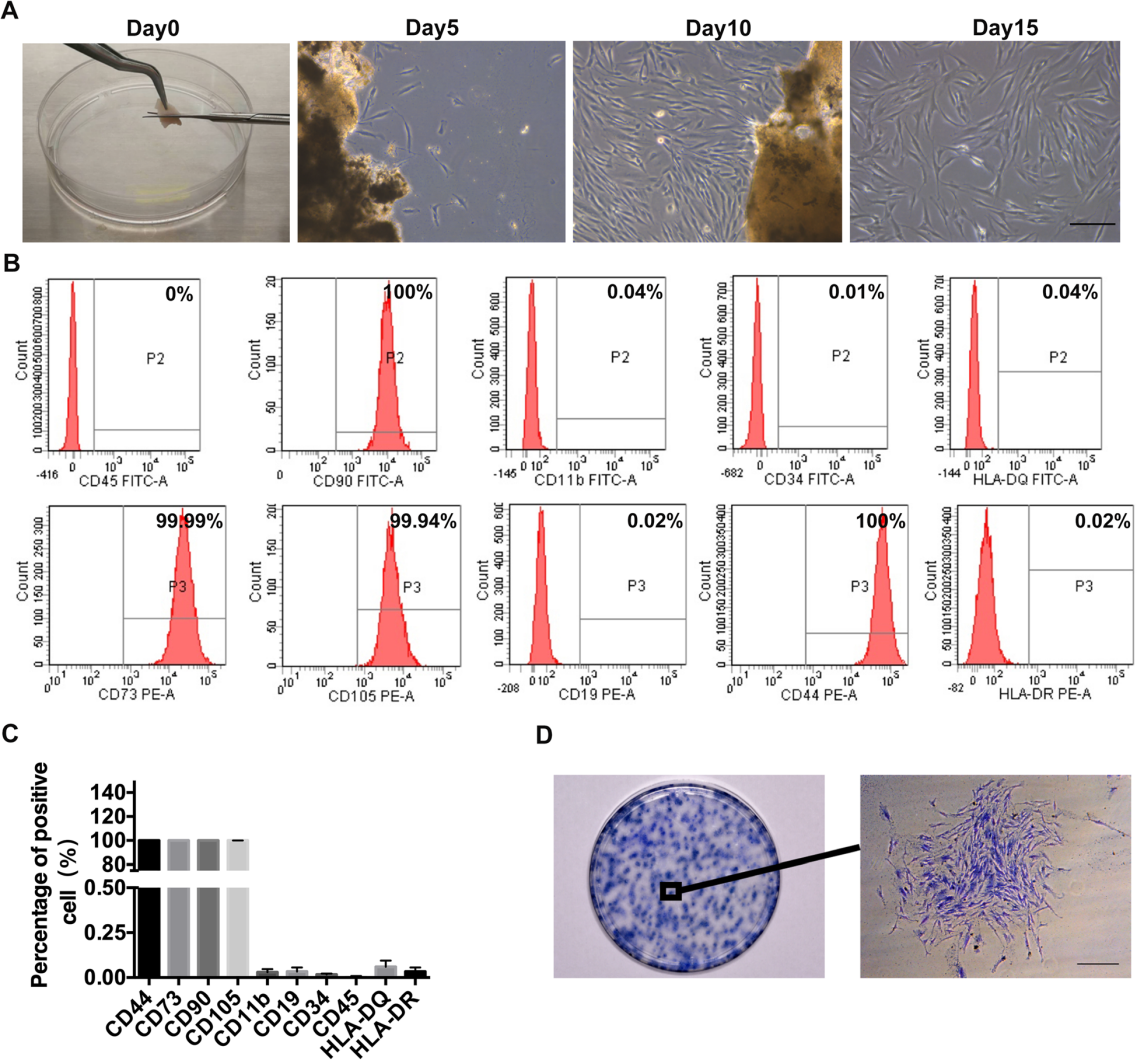
Isolation and identification of human GMSCs. **a** Typical morphology of fibroblast-like cells growing out from a gingival tissue fragment after culturing 5 days. When the cells reached 80% confluence from the passage 0 for about 10 days, they were subcultured and proliferated. The fibroblast-like cells were digested by trypsin and had high purity with a homogeneous spindle shape at passage 2 for 2 weeks. Scale bar, 500 μm. **b** and **c** Results of flow cytometry analysis were showed of the expression of cell surface markers related to mesenchymal stem cells (CD44, CD73, CD90, and CD105), hematopoietic stem cells (CD34 and CD45), or macrophage and B-cells (CD19, CD11b, HLA-DR, and HLA-DQ). CD, cluster of differentiation. *n* = 3. Error bars represent the SD. **d** The colonies were formed by human gingival mesenchymal stem cells (hGMSCs) at low seeding density for 2 weeks culture. Scale bar, 500 μm

After primary culture of hGMSCs derived from minced gingival tissues for about 5 days, there were short spindle fibroblast-like cells. The primary cells were digested and subcultured when they reached 80% confluence at 10 days. With the passaging culture, spindle-like cells achieved the advantage gradually and they had high purity with a homogeneous spindle shape at passage 2 after about half a month (Fig. 1a). Next, we assayed the surface marker expressions of hGMSCs by flow cytometry using antibodies of CD44, CD73, CD90, CD105, CD 34, CD45, CD19, CD11b, HLA-DQ, and HLA-DR. The results showed that hGMSCs highly expressed typical MSC surface markers (CD44, 100 ± 0.00%; CD73, 99.99 ± 0.01%; CD90, 100 ± 0.00%; and CD105, 99.93 ± 0.03%), but negative for hematopoietic stem cell surface markers or macrophage and B cells surface makers (CD34, 0.02% ± 0.01%; CD45, 0.00 ± 0.01%; CD19, 0.03 ± 0.02%; CD11b, 0.03% ± 0.02%; HLA-DR, 0.03 ± 0.023%; and HLA-DQ, 0.06 ± 0.03%). (Fig. 1b, c). To identify whether hGMSCs have colony-forming ability, cells were digested into single-cell suspensions at low density and 1 × 10^3^cells were seeded in 10-cm culture plate. The single hGMSC proliferated rapidly to form colony in next 14 days and presented fibroblastic morphology (Fig. 1d). Furthermore, we compared the osteogenic differentiation of human bone marrow mesenchymal stem cells (hBMSCs) which have been known for its perfect osteogenic differentiation for bone tissue regeneration [14], with hGMSCs in vitro. The alizarin red staining showed that the osteogenic differentiation potential of hGMSCs is weaker than that of hBMSCs (*p* < 0.05) (Additional file 1: Fig. S1).

### The effect of SB431542 treatment on the growth of hGMSCs

**Fig. 2:**
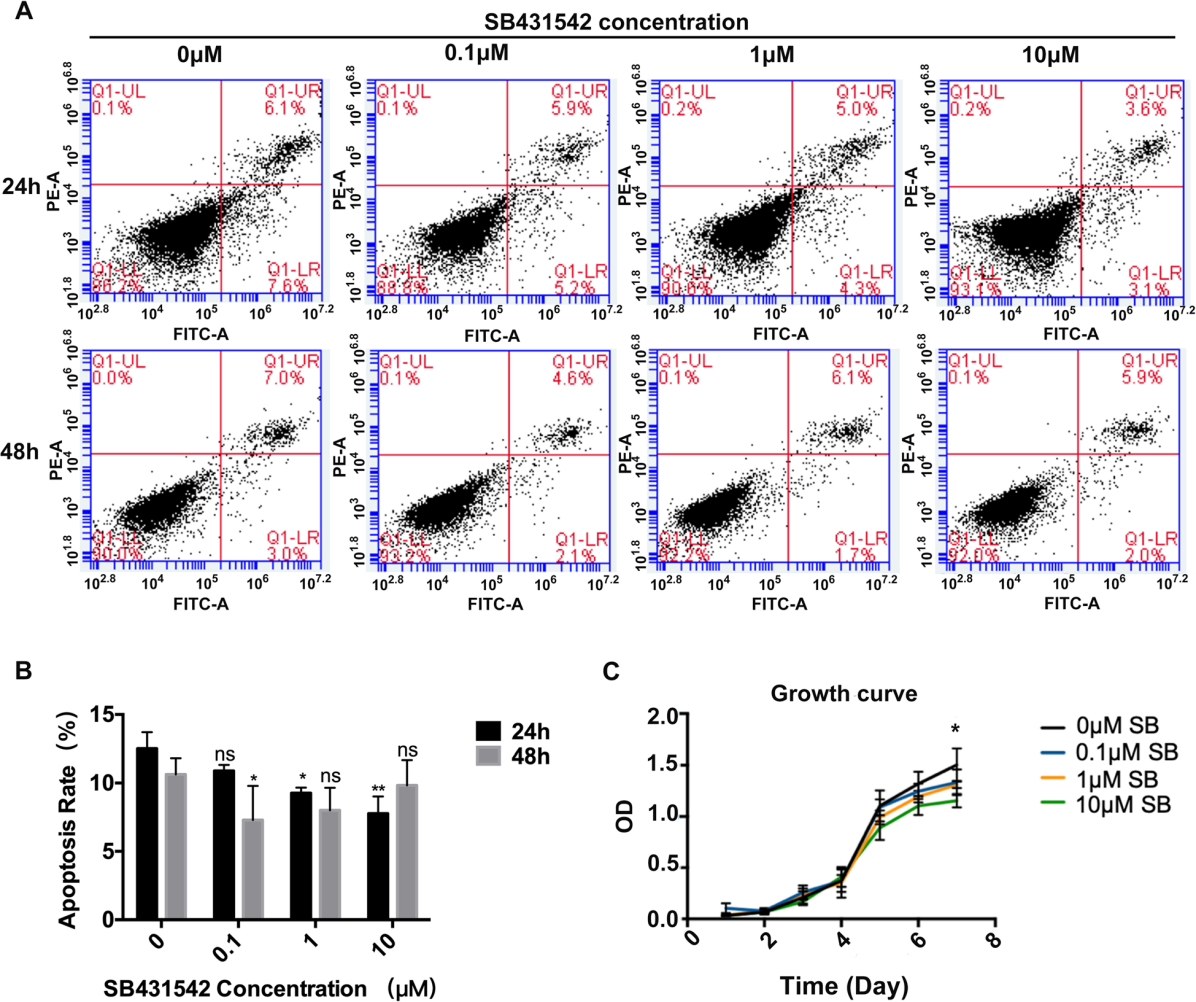
Effects of SB431542 on Growing status of hGMSCs. **a** and **b** Concentration-dependent apoptosis assay of hGMSCs with different concentration of SB4315342(0, 0.1, 1, and10μM) cultured for 24 h or 48 h. *n* = 3. **c** Cell growth curve of hGMSCs with different concentrations of SB4315342 (0, 0.1, 1, and10μM) by cell growth assay. Cell growth assay showed that the growth of group with 10 μM SB431542 was worse than the other groups at day7. *n* = 4. The results represent mean ± SD, ns, no statistically significant difference, *p* > 0.05, *p < 0.05, **p < 0.01, vs. 0 μM, one-way ANOVA with Tukey’s multiple comparison test

Next, we wanted to use a TGF-β signaling inhibitor SB431542 to enhance GMSCs osteogenesis. So firstly, we examined whether SB431542 affected the apoptosis and growth of hGMSCs. ANNEXIN V-FITC/PI apoptosis detection kit was used to assay the apoptosis of hGMSCs incubated with SB431542 at 0, 0.1, 1, and 10 μM. Interestingly, we found the apoptosis rates in 1 μM and 10 μM SB431542 groups were statistically lower than those in control group (0 μM SB431542 treatment) at 24 h (*p* < 0.05). At 48 h, 0.1 μM SB431542 group had a statistically lower apoptosis rate than control group (*p* < 0.05) (Fig. 2a, b). Although there were statistically differences in apoptosis rate among some groups, overall SB431542 treatment at indicated doses did not markedly affect the apoptosis of hGMSCs. Trypan blue staining showed SB431542 treatment at 0.1, 1, and 10μM did not affect the viability of hGMSCs at 24 h (*p* > 0.05) (Additional file 1: Fig. S2). The growth curve analysis by CCK-8 kit revealed 10 μM SB431542 markedly inhibited the proliferation of hGMSCs at 7 days (*p* < 0.05) (Fig. 2c). Based on these results, we used 1 μM SB431542 as maximum concentration in our following experiments.

### SB431542 treatment enables robust osteogenic differentiation of hGMSCs in vitro

**Fig. 3:**
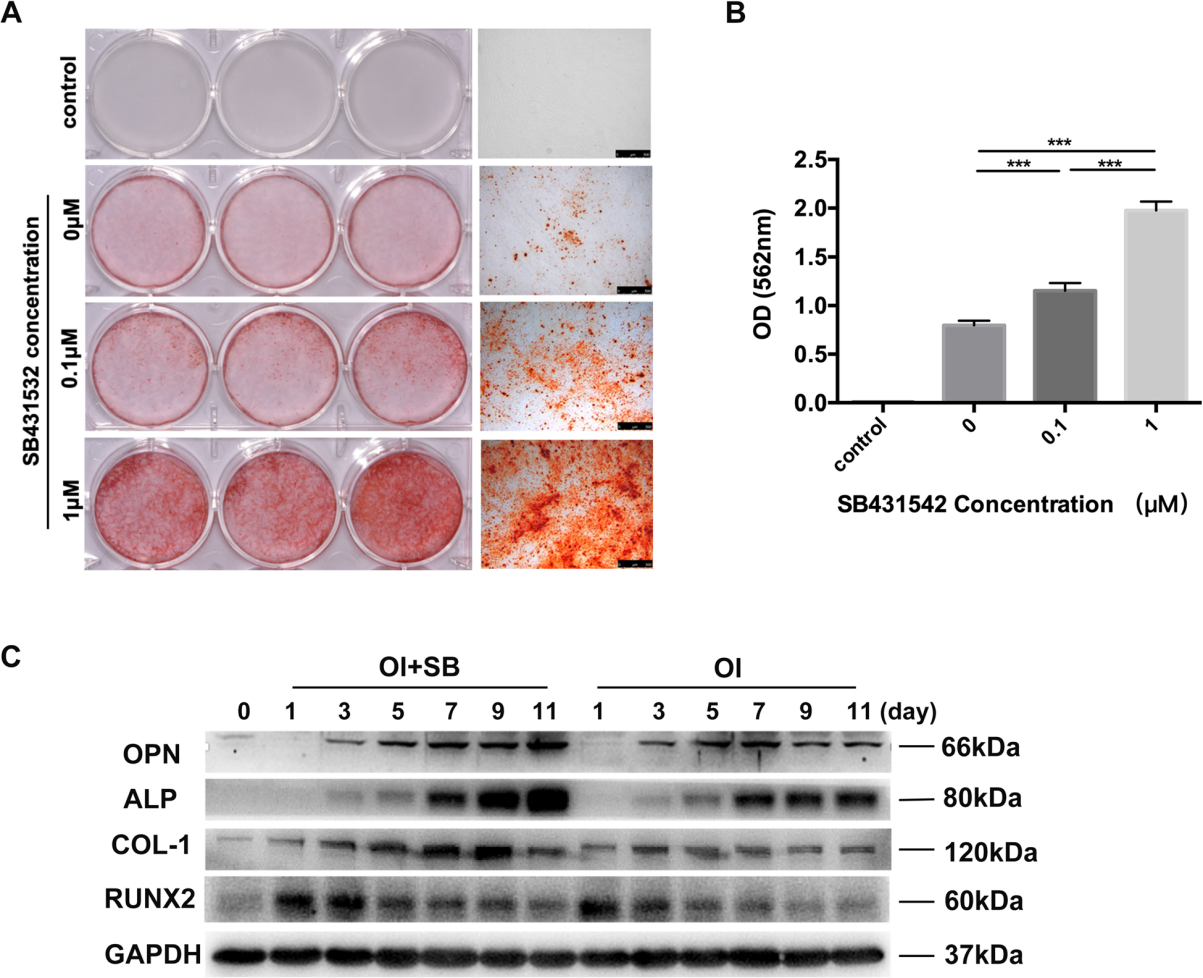
SB431542 treatment promotes osteogenic differentiation potential of hGMSCs. **a** hGMSCs formed mineralized nodules that stained positive for Alizarin Red following 21 days of osteogenic induction with various concentrations of SB4315342(0, 0.1, and1μM). hGMSCs showed improvement the capability of osteogenic differentiation as the concentration of SB4315342 increased. Scale bars, 500 μm. **b** Quantification of positive alizarin red staining showed that hGMSCs stimulated with SB431542 had significantly increased osteogenic differentiation capability compared with the other. *n* = 3. The results represent mean ± SD, ***p < 0.001, one-way ANOVA with Tukey’s multiple comparison test. **c** Western blotting analysis of osteoblastic differentiation-related protein in hGMSCs during osteogenic differentiation in the presence or absence of SB431542 (1 μM) at the indicated time points (days 0, 1, 3, 5, 7, 9, 11). GAPDH was used as a protein loading control. *n* = 3. OI, osteogenic induction medium; SB, 1 μM SB431542; D, day; ALP, alkaline phosphatase; COL-1, collagen type I; RUNX2, runt-related transcription factor 2; OPN, osteopontin

The alizarin red staining was performed to assay the calcified nodule formation in hGMSCs after osteogenic differentiation using specific differentiation medium. As shown in Fig. 3a, hGMSCs slightly formed the calcified nodules (red color) cultured with osteogenic differentiation medium for 21 days. Strikingly SB431542 treatment potentiates a robust formation of calcified nodules in a dose-dependent manner. Quantified data of positive alizarin red staining also confirmed that SB431542 treatment strikingly promoted osteogenic differentiation of hGMSCs in a dose-dependent manner (*p* < 0.05) (Fig. 3b). Next, we investigated spatiotemporal marker protein expressions related to osteogenic differentiation in hGMSCs during induction by differentiation medium. The osteogenic marker proteins including collagen type I (COL-1), alkaline phosphatase (ALP), osteopontin (OPN), and runt-related transcription factor 2 (RUNX2) were assayed by western blotting analysis (Additional file 1: Fig. S3), and the indicated proteins had respectively different spatiotemporal expression patterns during osteogenic induction. OPN protein expression started from day 4 to 11 and the expression level gradually increased. At day 1 during induction, COL-1 protein started to be expressed and then kept a stable expression level. The expression patterns of both ALP and RUNX2 proteins present a certain rhythm. Then, these protein expressions were assayed in hGMSCs after induction at indicated time points at presence of SB431542 (1 μM) or not (*p* < 0.05) (Fig. 3c). Comparing to the control group (without SB431542), SB431542 treatment markedly enhanced the overall expressions of OPN, ALP, COL-1, and RUNX2, especially ALP and COL-1. These results suggested that SB431542 treatment could dramatically accelerate ability of the osteoblastic differentiation and mineralization in hGMSCs in vitro.

### SB431542 treatment promotes subcutaneous osteogenesis of hGMSCs in nude mice

**Fig. 4:**
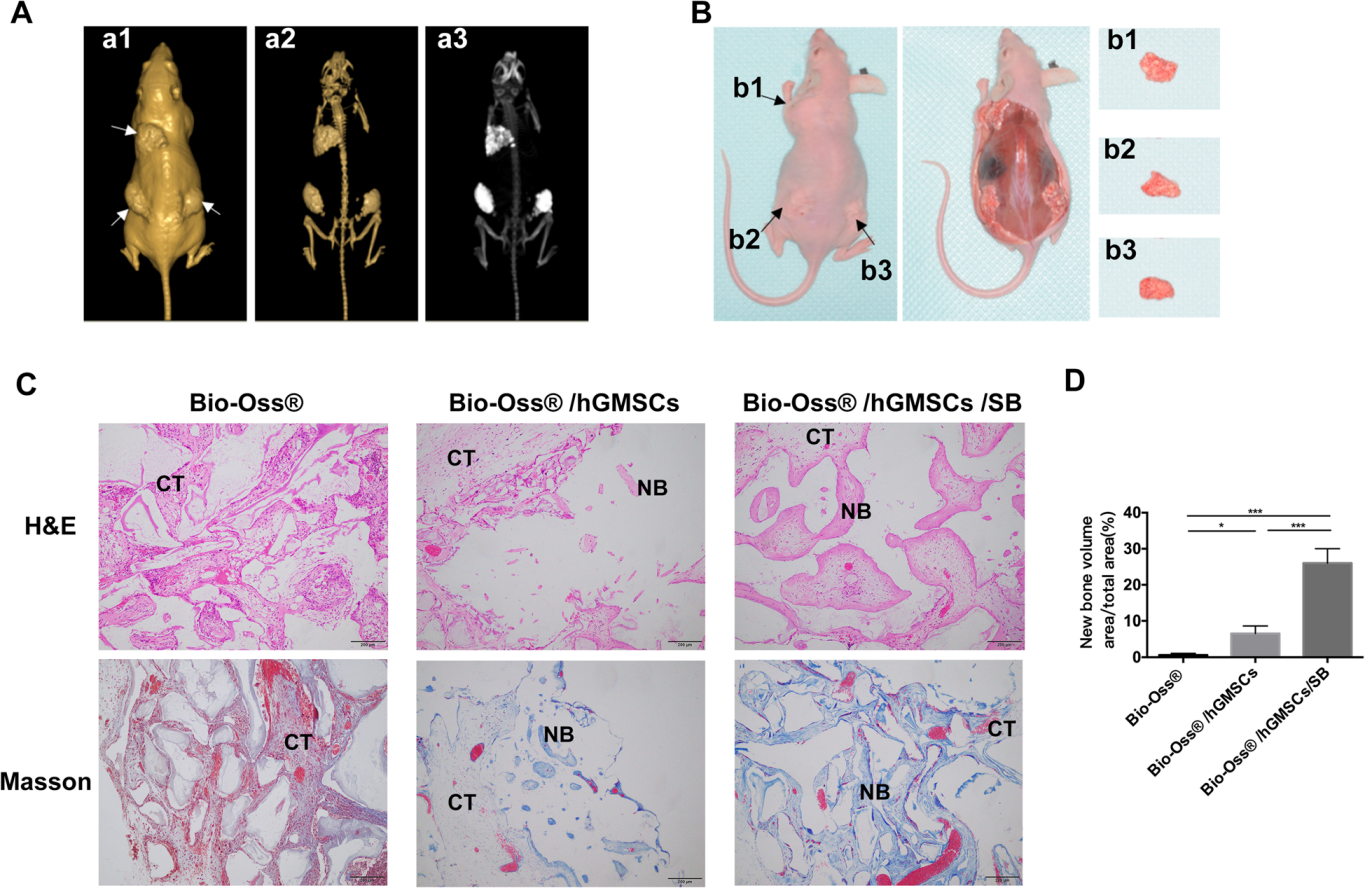
In vivo bone regeneration of hGMSCs in immunocompromised mice subcutaneously. **a** Three-dimensional (3D) visualization based on the CBCT images of mice.3D image creation of soft tissue (a1), 3D rendering of materials at mouse bone density (a2), and maximum intensity projection (MIP) full range visualization (a3) were created using the Software NNT viewer. CBCT, cone beam computed tomography. **b** Macroscopic view of allograft subcutaneous transplanted in immunocompromised mice skin at 12 weeks. The graft Bio-Oss® at upper left (b1), Bio-Oss®/hGMSCs at lower left (b2), and Bio-Oss®/hGMSCs/SB431542 at lower right (b3). **c** Representative images of specimen stained with H&E and Masson staining retrieved from immunocompromised mice after implantation for 12 weeks. CT, connective tissues; NB, new bone. Scale bars, 200 μm. H&E, Hematoxylin-Eosin; hGMSCs, human gingival mesenchymal stem cells; SB,1 μM SB431542. **d** Quantification of the new bone formation with H&E and Masson staining. *n* = 4 mice/group, 5 views each. *n* = 4 mice/group. Data represent mean ± SEM. *p < 0.05, ***p < 0.001, one-way ANOVA with Tukey’s multiple comparison test

Given a robust osteogenic differentiation ability induced by SB431542 in vitro, we wondered whether SB431542 also could induce such marked osteogenesis of hGMSCs in vivo. We translated scaffold Bio-Oss® with hGMSCs or hGMSCs/SB431542 into dorsal subcutaneous sites in mice and assayed osteogenesis at week 12 after implantation. The morphology of bone, soft tissue, and grafts were visualized by CBCT with the Software NNT viewer (Fig. 4a, b). Histological examinations of H&E and Masson staining showed that transplants with SB431542 displayed more new bone-like tissues including extracellular matrix (*p* < 0.05) (Fig. 4c, d). These in vivo data showed robust osteogenesis induction of SB431542 was very similar to those obtained in vitro.

### SB431542 elevates bone regeneration of autologous GMSCs to repair severe maxillary bone defects in minipigs

**Fig. 5:**
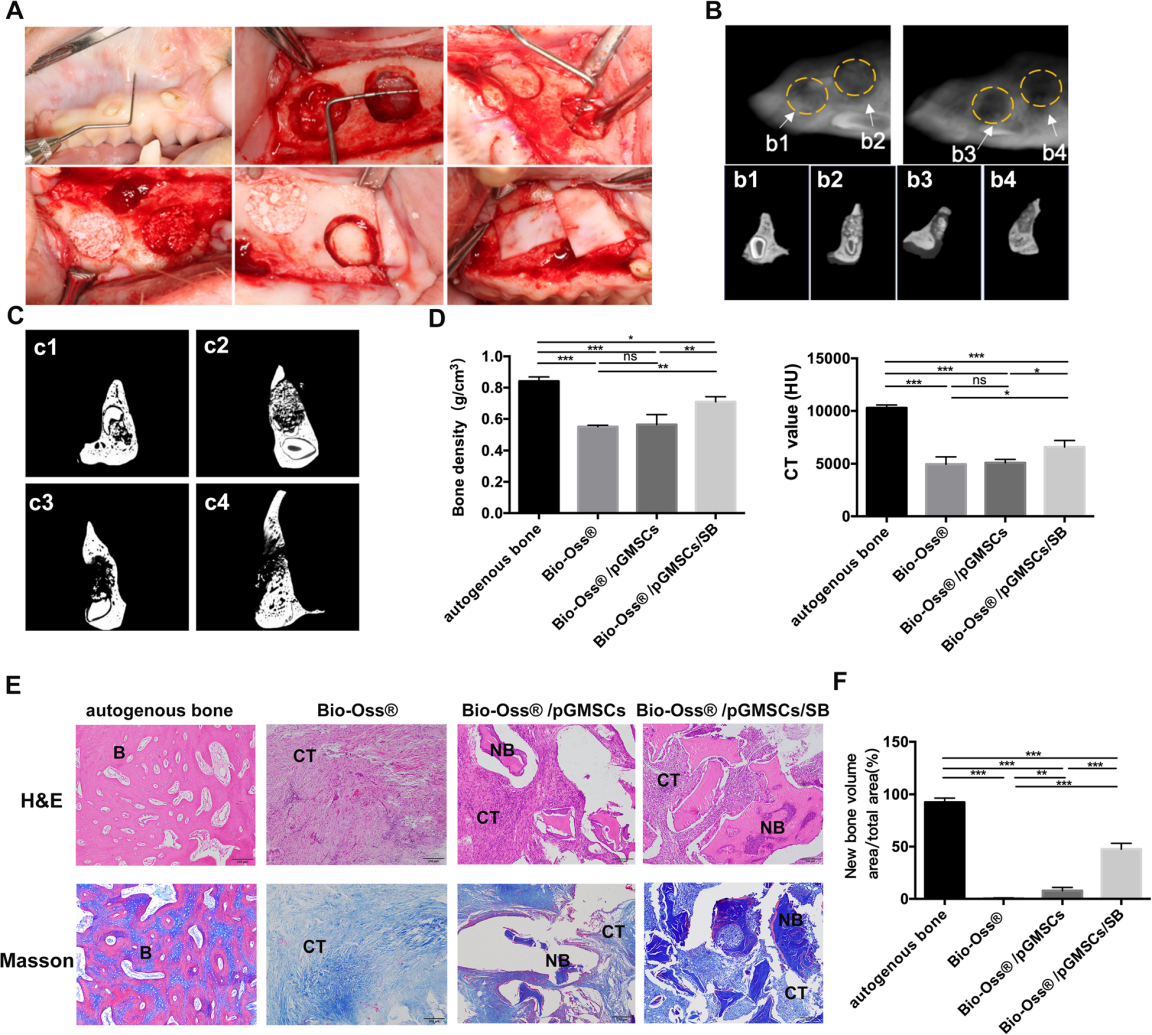
In vivo bone regeneration of autogenous pGMSCs in minipigs maxillary bone defects model. **a** Macroscopic view of surgical procedure of bone defect model in Minipigs. Defects measuring 12 mm in diameter and 5 mm in depth were prepared on each side of the maxillary. **b** CBCT images of grafts in maxillary bone defects at 2 months post-implantation. Maximum intensity projection full range visualization (Ac), were created using the Software NNT viewer. Images of 0.5-mm-thick free-cut specimens were generated across the four implants (yellow rings) individually. b1, autogenous bone blocks; b2, Bio-Oss®/pGMSCs/1 μM SB431542; b3, Bio-Oss®/pGMSCs; b4, Bio-Oss®. pGMSCs, pig gingival mesenchymal stem cells; CBCT, cone beam computed tomography. **c** Coronal images of Micro-CT show the different reparation effects of grafts. c1, autogenous bone blocks; c2, Bio-Oss®/pGMSCs/1 μM SB431542; c3, Bio-Oss®/pGMSCs; c4, Bio-Oss®. **d** The average CT value and bone density (BD) of new bone in the bone defects varied between the different groups analyzed by micro-CT. BD, bone density. *n* = 3, in each group. **e** Representative images of sections stained with Masson and H&E retrieved from minipigs after implantation for 2 months. B, mature bone; CT, connective tissues; NB, new bone. Scale bars, 200 μm. H&E, Hematoxylin-Eosin; **f** Quantification of the new bone formation by H&E and Masson staining. *n* = 3 pigs/group, 5 views each. *n* = 3 pigs/group. Data represent mean ± SEM. ns, no statistically significant difference *p* > 0.05, *p < 0.05, **p < 0.01, ***p < 0.001, one-way ANOVA with Tukey’s multiple comparison test

After we confirm the robust osteogenesis of SB431542 in vitro and in nude mice, we tried to use this effective osteogenic regeneration approach to achieve the goal of reconstructing the lost bone tissue. We established minipig maxillary bone defects model to further evaluate the therapeutic potential of GMSCs stimulated by SB431542 on large-volume bone repair. Worried of the potential risk of xenograft rejection using hGMSCs in minipigs, we decided to use autologous GMSCs derived from minipig (pGMSCs) in following big animal models. The pGMSCs were cultured as hGMSCs, and the morphology of pGMSCs, slightly rounded, were similar to hGMSCs (Additional file 1: Fig. S4A). Consistent with osteogenic differentiation result of hGMSCs in vitro, 1 μM SB431542 also remarkably enhanced osteogenesis of pGMSCs (Additional file 1: Fig. S4B, S4C). On the basis of results in vitro, next, the scaffold Bio-Oss® mixed with autogenous pGMSCs or pGMSCs/ SB431542 were implanted into the circular confined defect areas in minipigs (Fig. 5a). The autogenous bone implantation was set as positive control for bone regeneration.

At 2 months post-implantation, minipigs were sacrificed, and the specimens were harvested for CT (CBCT and Micro-CT) and histological analyses. In autogenous bone graft as golden standard for bone defect repair, CBCT and Micro-CT scan analyses showed the margin of the initial circular defects was not identifiable and initial circular defect was completely regenerated by the formation of mineralized tissue in sagittal and coronal view (Fig. 5b1, c1). In Bio-Oss®/pGMSCs/SB group, the initial defect areas showed high density with new bone trabeculae, closest to the autologous bone graft, compare to Bio-Oss® or Bio-Oss®/pGMSCs group. Besides, the margin of the initial circular defects was not clearly identifiable because of defect areas decreased and new bones well remodeled (Fig. 5b2, c2). In contrast, there were still obvious transmission and gaps surrounding the defects in other composite groups (Fig. 5c3, c4, d3, and d4). These results were confirmed by a quantitative analysis of BD and CT value, which showed the BD or CT value of Bio-Oss®/pGMSCs/SB group were higher compared to Bio-Oss® or Bio-Oss® /pGMSCs group in new bone formation (*n* = 3, in each group) (*p* < 0.05) (Fig. 5d). Moreover, as shown in histological analysis based on H&E and Masson staining, the autogenous bone-treated defects were almost filled with mature bones. And there was a large amount of new bone-like tissue and small amounts of connective tissue in Bio-Oss® /pGMSCs/SB treated defects, while only limited new formed-bone in Bio-Oss® /pGMSCs group. Instead, the single Bio-Oss®-treated defects were filled with almost fibrous tissue (Fig. 5e). As shown in Fig. 5f, the relative area of new formed-bone of Bio-Oss® /pGMSCs/SB group was 51.2 ± 5.54%, in the circular confined defects, higher than 11.6 ± 4.33% and 0.54 ± 0.45% in Bio-Oss® /pGMSCs group and Bio-Oss® group, respectively (*p* < 0.05). In summary, our preclinical study in vivo by CT and histological analyses revealed that SB431542 treatment could advance a bone regenerative process in repairing bone defect through stimulating a robust osteogenesis of GMSCs.

### SB431542-induced osteogenesis in hGMSCs by inhibiting canonical TGF-β signaling and activating BMP signaling

**Fig. 6:**
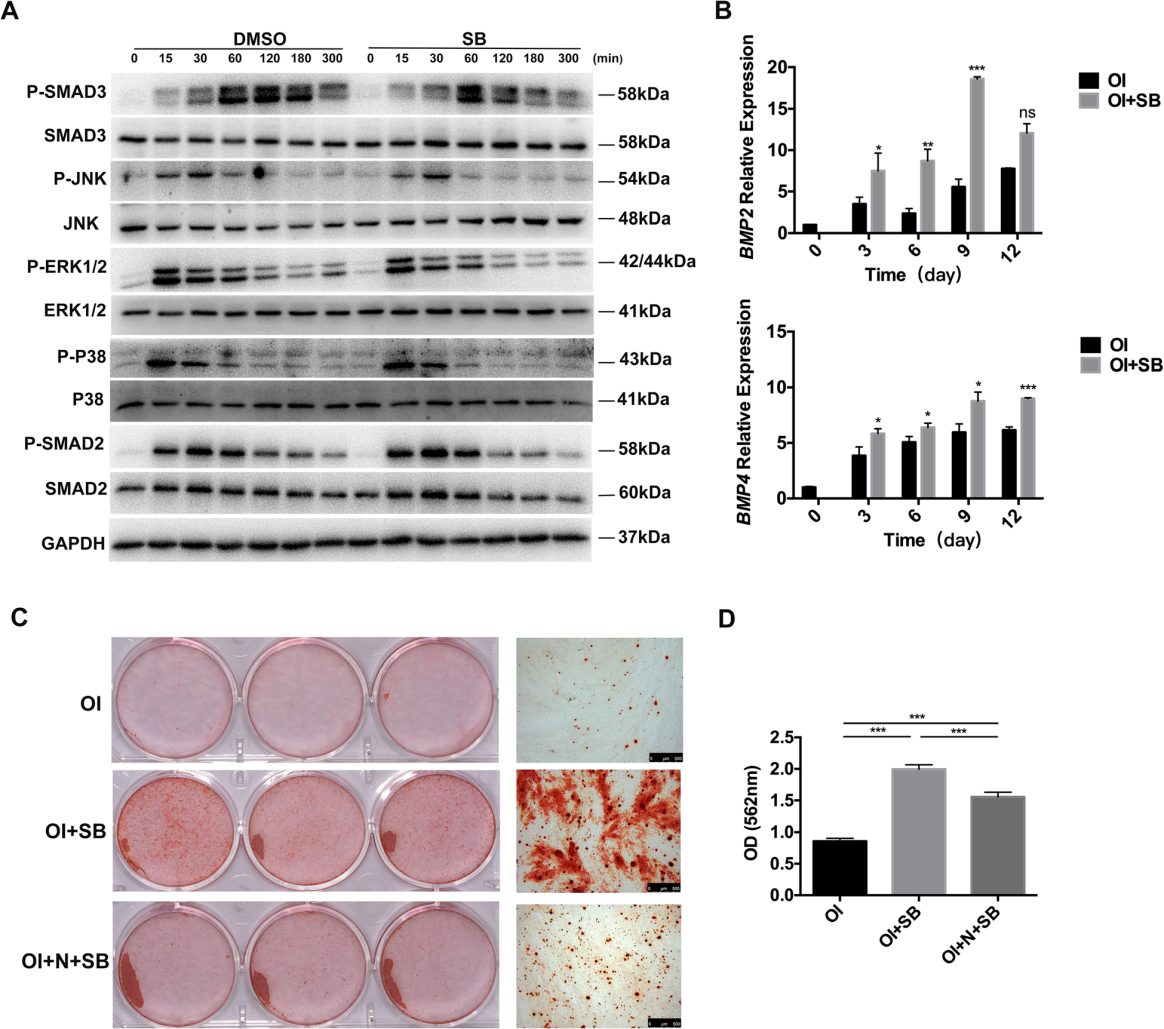
Effects of SB431542 on the TGF-β and BMP signaling in hGMSCs. **a** Western blotting analysis of phosphorylation protein levels of SMAD2, SMAD3, ERK1/2, JNK, and P38 with or without SB431542 (1 μM) at the indicated time points (0, 15, 30, 60, 120, 180, 300 min). GAPDH was used as a protein loading control. **b** mRNA expression of *BMP4* and *BMP6* were analyzed by qRT-PCR during osteogenic differentiation of hGMSCs treatment with or without SB431542 (1 μM) at a serious time points (0, 3, 6, 9, 12 day). The relative mRNA expression is normalized to *β-ACTIN*. Student’s *t* test. **c**, **d** Alizarin Red staining of hGMSCs (**c**) with quantification (**d**) after 21 days in osteogenic induction medium with both SB and noggin or SB alone. Osteogenic induction medium treatment as control. One-way ANOVA with Tukey’s multiple comparison test. *n* = 3. Data are means ± SEM. ns, no statistically significant difference *p* > 0.05, *p < 0.05, **p < 0.001, ***p < 0.001. Scale bars, 500 μm. SB, 1 μM SB431542; N, Noggin; OI, osteogenic induction medium; P, phosphorylation; min, minutes

To investigate the mechanisms of SB431542-induced osteogenesis in GMSCs, we finally assayed the difference of the mediators in TGF-β signaling pathway in hGMSCs at the presence of SB431542 (1 μM) or not. The western blotting analysis showed SB431542 treatment markedly inhibited the phosphorylation of SMAD3 at 120 and 180 min after incubation of osteogenic differentiation medium, compared with the control group (without SB431542 treatment). In contrast, SB431542 treatment did not affect the phosphorylation activation of ERK1/2, P38, SMAD2, and JNK (Fig. 6a). These results indicated SB431542 treatment specifically inhibited Smad3-dependent TGF-β signal pathway and induced the osteogenic differentiation in hGMSCs. Besides, as shown by qRT-PCR results, SB431542 treatment induced *BMP2* and *BMP4* upregulation relative to the control group (*p* < 0.05) (Fig. 6b). Furthermore, the ability of increasing calcified nodule formation by SB431542 treatment was blunted by a treatment of noggin, the BMP inhibitor (*p* < 0.05) (Fig. 6c, d). Collectively, these results exhibited that SB431542 competitively binds to TGF-β type I receptor, then inhibits SMAD3 phosphorylation, which activating BMP signaling by upregulating the gene expression of *BMP2* and *BMP4* finally resulting in a robust osteogenesis of hGMSCs. We supposed a schematic mechanism model of SB431542-induced osteogenesis in hGMSCs as follow. During the osteogenic differentiation of hGMSCs, TGF-β1 binds TGF-β type I receptor and causes phosphorylation of SMAD3, which in turn upregulates *BMP2* and *BMP4* and inactivates the target gene *RUNX2*.

## Discussion

In this preclinical study, we used a TGF-β signaling inhibitor SB431542 to induce osteogenic differentiation of GMSCs for repairing severe maxillofacial bone defect in big animal model. We found SB431542 treatment did not have adverse effect on apoptosis of hGMSCs and only high dose of SB431542 (10uM) slightly affected its proliferation. Under osteogenic medium culture, SB431542 treatment induced a robust osteogenesis of hGMSCs in a dose-dependent manner, representing striking formation of calcified nodules and expressions of osteogenesis-related protein markers such as COL-1, ALP, OPN, and RUNX2. Consistent with in vitro results, we found SB431542 treatment also enabled striking subcutaneous osteogenesis of hGMSCs in nude mice. In view of gingiva tissues being obtained and abundant from patient themselves for clinical use, hGMSCs can be postulated to be an easily accessible and prominent source for the seeding cells. Then, minipig maxillary bone defect model was established to evaluate the therapeutic potential of GMSCs stimulated by SB431542 on large-volume bone repair. The bone defect repair experiment showed SB431542 could advance a bone regenerative process in repairing severe bone defect through stimulating a robust osteogenesis of GMSCs. Finally, we found that SB431542 treatment induced the osteogenic differentiation of hGMSCs by inhibiting Smad3-dependent TGF-β signal pathway.

Reconstruction of complex skeletal defect still is a huge challenge, especially maxillofacial bone defect restoration. SCBT is a very promising approach to solve these problems. A variety of MSCs have been well investigated as a good cell source to be applied to bone regeneration and tissue engineering because of their osteogenic differentiation potential [31]. Up to now, BMSCs are considered as main source of autologous seeding cells for bone regeneration in clinic. But the amount of BMSCs in the bone marrow is extremely low which limits the isolation and yield harvest. Besides, the potential of proliferation and differentiation of BMSCs are affected by the age of the donors and culture passages [32]. In this study, we isolated MSCs from adult gingival tissues. The hGMSCs were easily isolated from discarded gingival tissues in the dental clinic without extra invasive operation, compared with hBMSCs. And a study about hGMSCs properties demonstrated that, compared to BMSCs, GMSCs show a faster proliferation rate and maintain stable phenotype, karyotype, and telomerase activity in long-term cultures [18]. The hGMSCs expressed high levels of surface markers including CD44, CD73, CD90, and CD105, not expressing CD34, CD11b, HLA-DR, and HLA-DQ. These cells had a good colony-forming ability and potential of osteogenic, adipogenic, and chondrogenic differentiation. The hGMSCs had similar classical characteristics with general MSCs and were a more accessible cell source for clinical use. Thus, the adult human gingival tissue could be considered as one of the most promising seeding cell sources for bone tissue engineering.

Until now, the autologous bone transplantation is considered as gold standard for maxillofacial bone defect repair. In this treatment, bone tissue removed from patient own was transplanted to bone defect as an autologous bone filling stuff [33]. However, there are some shortcomings using this method for severe bone defects. For example, the amount of bone material for cleft lip and palate patients is often not sufficient to stuff wide fissures of alveolar cleft. Solving bone increment for filling the bone defect is the key in oral clinical practice for patients with large maxillofacial defect. Although various MSCs have been widely investigated their potential in bone tissue engineering, their osteogenic differentiation was very limited without extra interventions in vivo and in vitro. Especially, the niche in bone defect goes against the bone regeneration because of inflammatory response, ischemia, oxidative stress, and neurotrophic factor deprivation. In this study, we used a small molecule SB431542, a TGF-β signal inhibitor, to induce a robust osteogenesis of hGMSCs in a dose-dependent manner, without adverse effects on the growth and apoptosis in vitro. Next, we investigated whether SB431542 treatment also could enable striking ontogenesis in vivo. Results showed SB431542 treatment induced marked subcutaneous osteogenesis of hGMSCs in nude mice, consistent with data in vitro. We supposed that SB431542 could solve the difficulty of bone increment for severe maxillary bone defect. Then, minipig maxillary bone defect model was established, and GMSCs combined with Bio-Oss® were transplanted into injured sites at the presence of SB431542 or not. Bio-Oss® was a general bone repair material and act as a scaffold for GMSCs in our models [34]. We found SB431542 treatment had a satisfactory outcome, which is close to the gold standard group of autologous bone transplantation. These results indicated a great potential for GMSCs combination with SB431542 in treating severe maxillary bone defect in clinic.

TGF-β signal pathway plays a very important role in bone metabolism and physiology [35, 36]. SB431542, acting as a TGF-β signal inhibitor, have been used in bone reconstruction and regeneration. Senarath-Yapa et al. reported SB431542 promoted the repair of calvarial skeletal defect in mice by enabling endogenous bone regeneration [2]. This report found that bone morphogenetic protein (BMP) signaling was activated in dura matter cells and osteoblasts participating in repairing calvarial defect. In addition, after inhibiting TGF-β signaling, the higher levels of BMP2 in cells upregulated the expression of inhibitory SMAD6, which in return contained excessive BMP2 signaling during bone regeneration process. Another study showed that SB431542 treatment could induce the commitment of periodontal ligament cells into hard bone cells in a BMP2-dependent manner in vitro. Without additional BMP2 protein into medium, SB431542 treatment failed to induce the expressions of oesteogenic genes and the formation of mineralized nodule in periodontal ligament cells [25]. Therefore, it is speculated that TGF-β, as an important signaling in human, plays an extremely complex regulation effects on bone regeneration and development. Namely, the TGF-β signaling inhibition by SB431542 has discrepant mechanism in regulating osteogenic differentiation of cells depending on their context. Therefore, it is speculated that TGF-β signaling, as an important cytokine in human body, has an extremely complex regulation effect. The mechanism of SB431542 inhibiting TGF-β signaling to regulate osteogenic differentiation of different cells is not consistent. In our study, we found that SB431542 treatment induced striking formation of calcified nodules and expressions of osteogenesis-related protein markers such as COL-1, ALP, OPN, and RUNX2 during the robust osteogenesis of hGMSCs. In addition, SB431542 inhibited the phosphorylation level of SMAD3 in TGF-β signaling pathway in osteogenic differentiation process. TGF-β1 binds TGF-β type I receptor and causes phosphorylation of Smad3, which in turn translocates into nucleus and inactivates the target gene RUNX2. RUNX2 is an important transcription factor for regulating osteogenic gene expressions. We supposed a potential mechanism of SB431542-induced osteogenesis in hGMSCs. SB431542 competitively binds to TGF-β type I receptor and inhibits the phosphorylation of Smad3 of TGF-β signaling, upregulating the gene expression of RUNX2 which induced osteogenesis-related gene expression. One limitation of this study is that it need further study to investigate how the decreased Smad3 activity regulating the RUNX2 and other osteogenic proteins during osteogenesis of hGMSCs induced by TGF-β signaling inhibition by SB431542. In addition, to further demonstrate the efficacy of the proposed approach as a viable alternative to BMSCs-based bone regeneration, in further experiment, BMSCs should be included as control to determine whether TGF-β inhibitor treatment of GMSCs adequately rescue the osteogenesis to the level of BMSCs.

## Conclusion

In conclusions, this study provides the first evidence to demonstrate that autologous GMSCs treated with a TGF-β signaling inhibitor SB431542 successfully repair minipig severe maxillofacial bone defects. This preclinical study shows a promising large bone regeneration therapeutic potential of autologous easy accessed GMSCs induced by SB431542 in clinic settings. Given a high osteogenic differentiation potential, a further experiment with BMSCs as control is designed to determine whether TGF-β inhibitor treatment of GMSCs is sufficient to rescue new bone formation to BMSCs level.

## Additional file

Additional file 1: **Figure S1.** Comparison of osteogenic differentiation potential between hGMSCs and hBMSCs. **Figure S2.** Cell viability assay. **Figure S3.** Western blotting analysis of osteoblastic differentiation-related protein. **Figure S4.** Pig gingival mesenchymal stem cells (pGMSCs) in vitro*.* **Table S1.** Primer sequences in qRT-PCR. (DOCX 3545 kb)

## Abbreviations

ALP: Alkaline phosphatase
BD: Bone density
BMP: Bone morphogenetic protein
CBCT: Cone beam computed tomography
CD: Cluster of differentiation
COL-1: Collagen type I
H&E: Hematoxylin-Eosin
hBMSCs: Human bone marrow mesenchymal stem cells
hGMSCs: Human gingival mesenchymal stem cells
MSCs: Mesenchymal stem cells
OCN: Osteocalcin
OI: Osteogenic induction medium
OPN: Osteopontin
pGMSCs: Pig gingival mesenchymal stem cells
qRT-PCR: Quantitative real time polymerase chain reaction
RUNX2: Runt-related transcription factor 2
SB: SB431542
SCBT: Stem cell-based therapy
TGF-β: The transforming growth factor beta
