# Supplementary material for: Small molecule inhibitor of TGF-β signaling enables robust osteogenesis of autologous GMSCs to successfully repair minipig severe maxillofacial bone defects
Source: Stem Cell Res Ther. 2019 Jun 13;10:172. doi: 10.1186/s13287-019-1281-2 (PMC6567469; doi:10.1186/s13287-019-1281-2)
Supplement: Supplementary file 1 — Figure S1. Comparison of osteogenic differentiation potential between hGMSCs and hBMSCs. Figure S2. Cell viability assay. Figure S3. Western blotting analysis of osteoblastic differentiation-related protein. Figure S4. Pig gingival mesenchymal stem cells (pGMSCs) in vitro. Table S1. Primer sequences in qRT-PCR. (DOCX 3545 kb) [file 13287_2019_1281_MOESM1_ESM.docx]

**Small Molecule Inhibitor of TGF-β Signaling Enables Robust Osteogenesis of Autologous GMSCs to** **Successfully Repair Minipig Severe** **Maxillofacial Bone Defects**

Anyuan Shi, Aerali Heinayati, Dongyu Bao, Huifen Liu, Xiaochen Ding, Xin Tong, Liudi Wang, Bin Wang, Haiyan Qin

**Supplemental Figure 1**

**
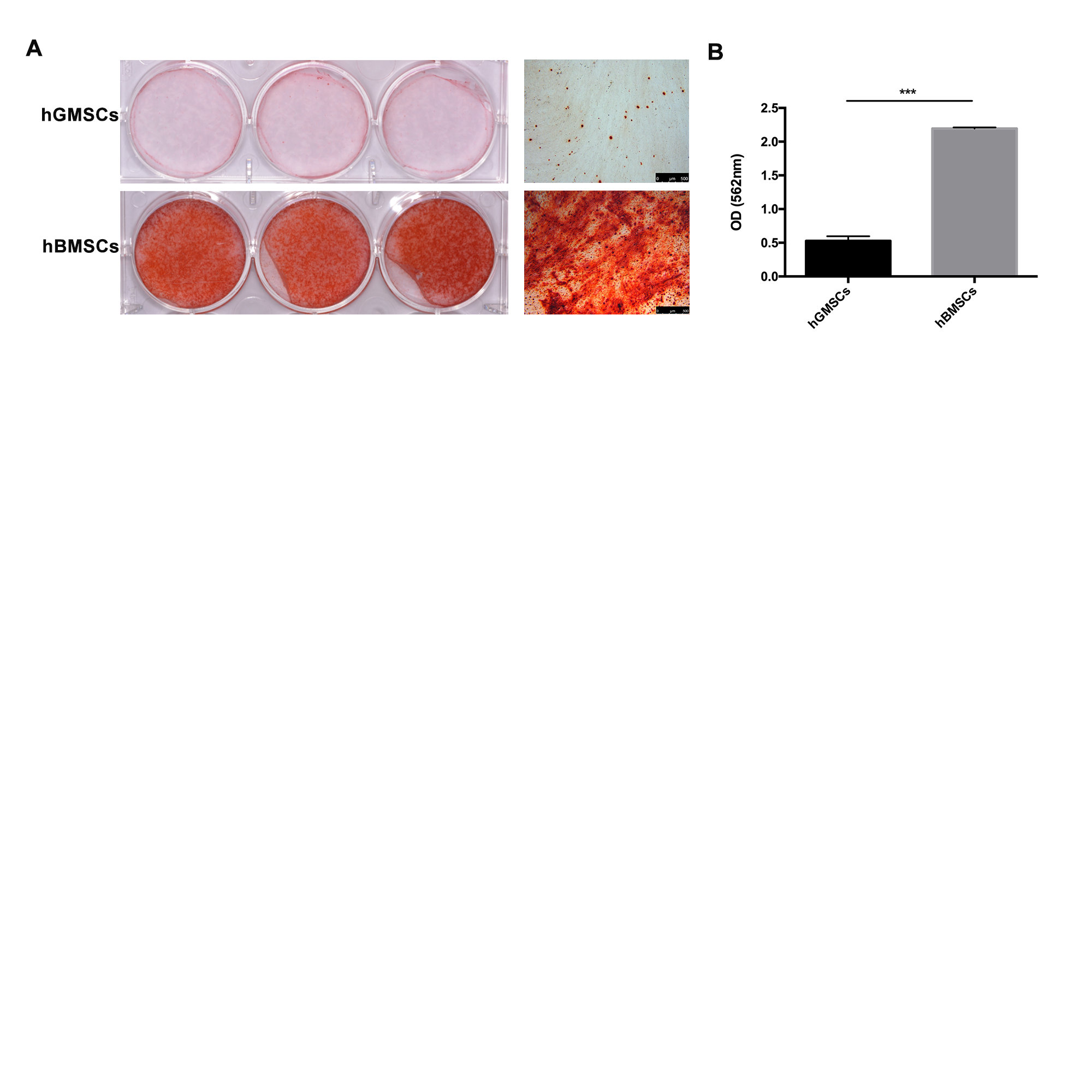
**

**Figure S1.** **Comparison of osteogenic differentiation potential between hGMSCs and hBMSCs.**

(A) human gingival mesenchymal stem cells (hGMSCs) and human bone marrow mesenchymal stem cells(hBMSCs) were treated with osteogenic induced medium (OI). Mineralized nodules were formed that stained positive for alizarin red following 21 days of osteogenic induction, respectively.

(B) quantification of mineralized nodules by alizarin red staining with 10 % cetylpyridinium chloride.

Scale bars, 500μm. n=3. Data are means ± SEM. ****p* < 0.001, student’s t test.

**Supplemental Figure2**

**
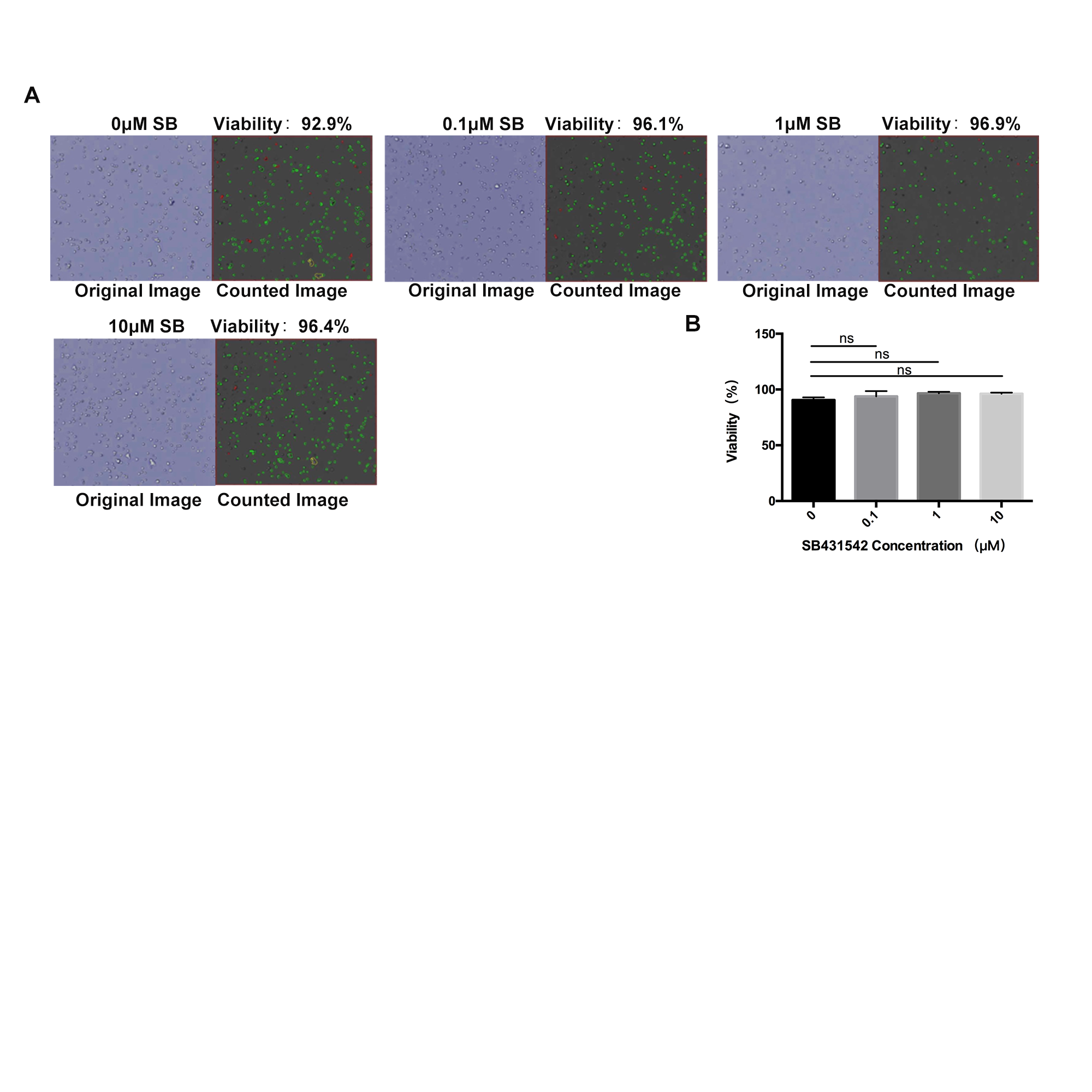
**

**Figure S2**. **Cell viability assay**.

(A and B) The result of trypan blue stain(A) with quantification assay(B), SB concentration has no damage on cell viability after incubation 24 hours. n = 3. Data are means ± SEM. ns, no statistically significant difference (*p* >0.05) vs 0μM, one-way ANOVA with Tukey’s multiple comparison test. SB, SB431542.

**Supplemental Figure 3**

**
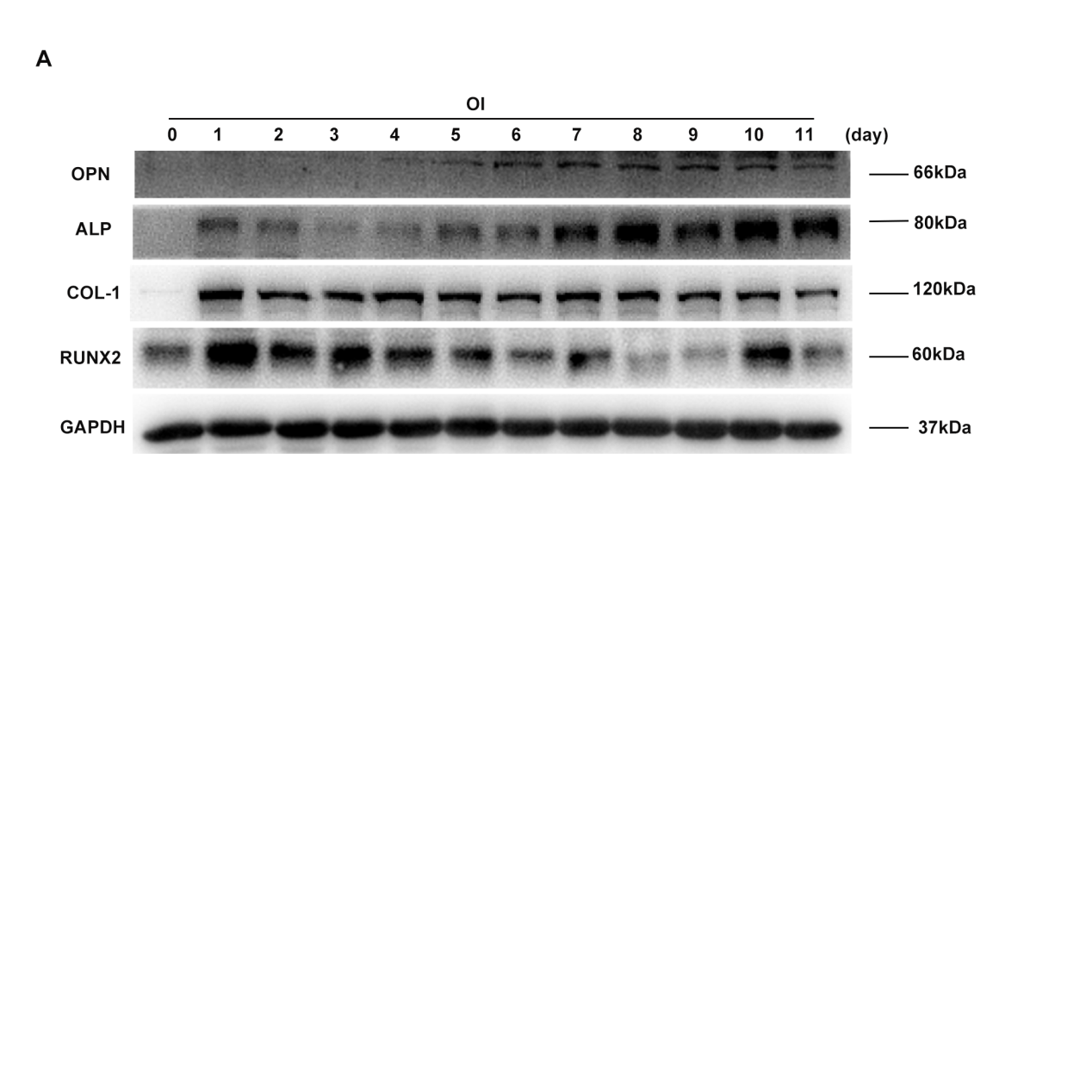
**

**Figure S3. Western blotting analysis of osteoblastic differentiation-related protein.**

(A)Samples that osteoblastic differentiation-related proteins (ALP, OPN, COL-1, RUNX2) were collected from hGMSCs of osteogenic induction for 11 days per day. GAPDH was used as a protein loading control.

n=3. OI, osteogenic induction medium; ALP, alkaline phosphatase; COL-1, collagen type I. RUNX2; runt-related transcription factor 2; OPN, osteopontin; hGMSCs, human gingival mesenchymal stem cells.

**Supplemental Figure 4**


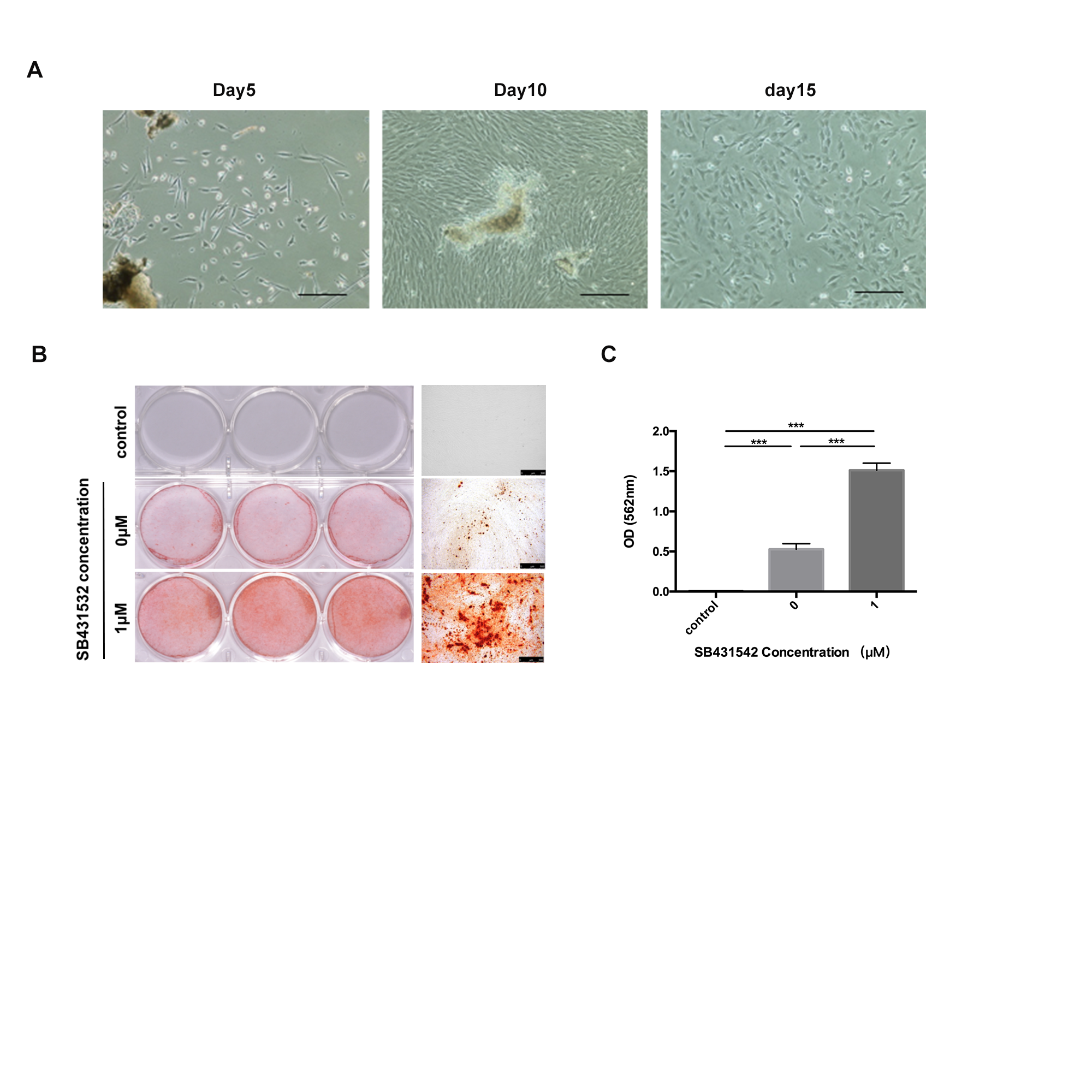


**Figure S4**. **Pig gingival mesenchymal stem cells (pGMSCs) *in vitro.***

(A) Isolation and culture of pGMSCs. The slightly rounded morphology of pGMSCs is slightly different from that of hGMSCs. Scale bars, 500 μm. pGMSCs, pig gingival mesenchymal stem cells.

(B and C) Alizarin Red staining of pGMSCs (B) with quantification (C)after 21 days in osteogenic induction medium with or without SB. Basal culture medium as control n =3. Data are means ± SEM. ****p* < 0.001, one-way ANOVA with Tukey’s multiple comparison test. Scale bars, 500μm.

| \| Gene（human） \| Primer Sequences (5’ to 3’) \| Product Length (bp) \| NCBI Reference Sequence \| \| --- \| --- \| --- \| --- \| |
| --- | --- | --- | --- | --- |
| *β-ACTIN* F：AGCACAGAGCCTCGCC 229 NM_001101.5   \| R: CTCGTCGCCCACATAGGAAT \| \| --- \|   *BMP2*  F：GAATAACTTGCGCACCCCAC 306 NM_001200.4  R: CCACCATGGTCGACCTTTAGGA  *BMP4* F：TAGCAAGAGTGCCGTCATTCC 109 NM_001202.6  R：GCGCTCAGGATACTCAAGACC |

**Supplemental Table**

**Table S1. Primer sequences in qRT-PCR**.

F, forward; R, reverse. *BMP2: bone morphogenetic protein 2; BMP4: bone morphogenetic protein 4.*
